# 25-Hydroxyvitamin D and Vitamin D Binding Protein Levels in Patients With Primary Hyperparathyroidism Before and After Parathyroidectomy

**DOI:** 10.3389/fendo.2019.00171

**Published:** 2019-03-27

**Authors:** Xiangbing Wang, Zhifeng Sheng, Lingqiong Meng, Chi Su, Stanley Trooskin, Sue A. Shapses

**Affiliations:** ^1^Divisions of Endocrinology, Metabolism, and Nutrition, Departments of Medicine and Surgery, Rutgers-Robert Wood Johnson Medical School, New Brunswick, NJ, United States; ^2^Institution of Metabolism and Endocrinology, The Second Xiangya Hospital, Central South University, Changsha, China; ^3^Department of Nutritional Sciences, Rutgers University, New Brunswick, NJ, United States; ^4^Divisions of General Surgery, Departments of Medicine and Surgery, Rutgers-Robert Wood Johnson Medical School, New Brunswick, NJ, United States

**Keywords:** vitamin D binding protein, vitamin D deficiency, parathyroid hormone, calcium metabolism, hyperparathyroidism, parathyroidectomy

## Abstract

**Objective:** To evaluate vitamin D binding protein and free 25-hydroxyvitamin D [25(OH)D] levels in healthy controls compared to primary hyperparathyroidism (PHPT) patients, and to examine PHPT before and after surgery.

**Methods:** Seventy-five PHPT patients and 75 healthy age, gender, and body mass index (BMI) -matched control subjects were examined. In addition, 25 PHPT patients underwent parathyroidectomy and had a 3-month follow up visit. Levels of total and free 25(OH)D, DBP, and intact parathyroid hormone (iPTH) were determined before and 3 months after surgery.

**Results:** There was no significant difference in age and BMI between PHPT patients and controls. Levels of 25(OH)D and DBP were lower in PHPT patients compared to controls (*p* < 0.01). There was no significant difference in calculated free and bioavailable 25(OH)D levels between PHPT patients and controls. Calcium and iPTH levels decreased to normal but DBP and DBP-bound-25(OH)D increased (*P* < 0.001) after parathyroidectomy. Levels of DBP were inversely correlated with iPTH (*r* = −0.406, *P* < 0.001) and calcium levels (*r* = −0.423, *P* < 0.001).

**Conclusion:** Serum DBP levels were lower in patients with PHPT and parathyroidectomy restored DBP levels. We suggest that lower DBP levels is one of contributing mechanisms of low total 25(OH)D in PTHP patients and the total 25(OH)D levels might not reflect true vitamin D status in PHPT patients.

## Introduction

Total 25(OH)D level has been recognized as an optimal indicator of vitamin D nutrition status, and lower 25(OH)D concentration is usually considered as vitamin D deficiency or insufficiency in clinical practice. Low total 25(OH)D concentration, which is common in PHPT patients, is associated with the severity of the disease and high parathyroid adenoma weight (1–3). Low 25(OH)D levels also exist in many chronic conditions such as end-stage liver disease and nephrotic syndrome, and in critical illness where intact parathyroid hormone levels are not elevated (4, 5). The majority of circulating 25(OH)D tightly bound to DBP, with a smaller amount (10–15%) bound to albumin. Less than 1% of circulating vitamin D metabolites exists in a free, unbound form (5). The variations in the 25(OH)D levels in these conditions result from variations in the binding of 25(OH)D to DBP (4).

Previous studies showed that DBP are lower in PHPT patients compared with age, BMI, and gender matched (6–8) or genetic background matched controlled subjects (7). It is unclear how DBP is regulated in and if the elevated iPTH plays a role in PHPT, or if DBP simply a biomarker of circulating 25(OH)D. Since the majority (>85%) of circulating 25(OH)D is bound to DBP, we suggest that decreased DBP might be one of mechanisms of low total 25(OH)D levels in PHPT patient (8). The causes of lower DBP concentration in the serum of PHPT patients remained unknown. We hypothesize that the elevated iPTH or calcium levels inhibit DBP production in the liver of PHPT patient. In our current study, we investigated the effects of lower calcium and iPTH levels by parathyroidectomy on DPB in PHPT patients. We also compared levels of 25(OH)D, DBP, and calculated free and bioavailable 25(OH)D in patients with PHPT with normal controls. The aim of this study is to investigate the effects of parathyroidectomy on DBP and DBP-bound 25(OH)D levels in PHPT patients.

## Methods

### Study Subjects

Seventy-five PHPT patients (61 Caucasians, eight African American, four Asians, and two Hispanic Americans) were seen at the Endocrinology and General Surgery clinics of Robert Wood Johnson University Hospital from January 2010 to December 2016 at prior to treatment. Most of the patients were relatively asymptomatic with less severe PHPT profile (9). The inclusion criteria were: (1) a serum calcium level >10.6 mg/dL (8.6–10.4 mg/dl) and intact PTH (iPTH) >66 pg/mL (15–65 pg/mL), (2) age 20–80 years, and (3) 24-h urine calcium >100 mg (100–300 mg/24 h) with fraction excretion of calcium >0.01. The exclusion criteria were: (1) hormone replacement therapy or contraceptive pills, (2) hepatic dysfunction, or (3) renal dysfunction, and (4) BMI >40 (kg/m^2^). Seventy-five age, gender, and BMI-matched healthy volunteers (62 Caucasians, eight African Americans, four Asians, and one Hispanic American) (10) from the community were included as controls after a multistep screening process and did not take contraceptive pills. The healthy controls took 400 IU vitamin D supplement. Supplemental vitamin D intake in patients before surgery is not known. Twenty-five PHPT patients (seven males and 18 females) underwent parathyroidectomy (PTX) monitored by intro-operative iPHT levels and were examined at 3 months during their follow-up visit after surgery. All minimally invasive PTX were done by one surgeon and all patients were advised to take 0.25 mcg calcitriol for 1–2 weeks and 1,000–2,000 IU vitamin D for 1–3 months after PTX to prevent hypocalcemia as standard post-operative clinical care (11). All subjects and patients signed an informed consent and the use of human subjects in this study was approved by the IRB at Rutgers University.

### Sample Collections and Assays

Venous blood samples were collected from patients and controlled subjects after a 12-h overnight fast. Twenty-five PHPT patients had parathyroid surgery and finished 3 months' post-surgery follow up visit. Serum was separated and stored at −70 C for measurement of 25(OH)D and DBP levels. Intact-PTH, serum calcium, and albumin were determined by commercial laboratories. The laboratory uses both internal and external standards, and also participated in the international Vitamin D External Quality Assessment Scheme to ensure the quality and accuracy of the 25(OH)D analysis and serum 25(OH)D levels (radioimmunoassay; DiaSorin) CV < 12.5%). DBP levels in serum were determined using a commercial polyclonal ELISA kit (ALPCO, Salem, NH). The intra- and inter-assay coefficients of variation are 5.0 and 12.7%, respectively. Free, bioavailable, albumin-bound and DBP-bound 25(OH)D concentrations were calculated using equations adapted from Bikle et al. (12).

### Statistical Analyses

Results are expressed as mean ± SD. Shapiro-Wilk test was used to check normality. Two-tailed Student's *t*-test and Wilcoxon Rank Sum test were used to compare values between groups with normally and non-normally distribution, respectively. Changes before and after parathyroidectomy were compared with a paired Student's *t*-test. Correlation coefficients and linear regression were used to assess relationships between variables. A *P* < 0.05 was defined as the level of significance. Statistical analysis was performed with SAS v9.4.

## Results

**Figure 1:**
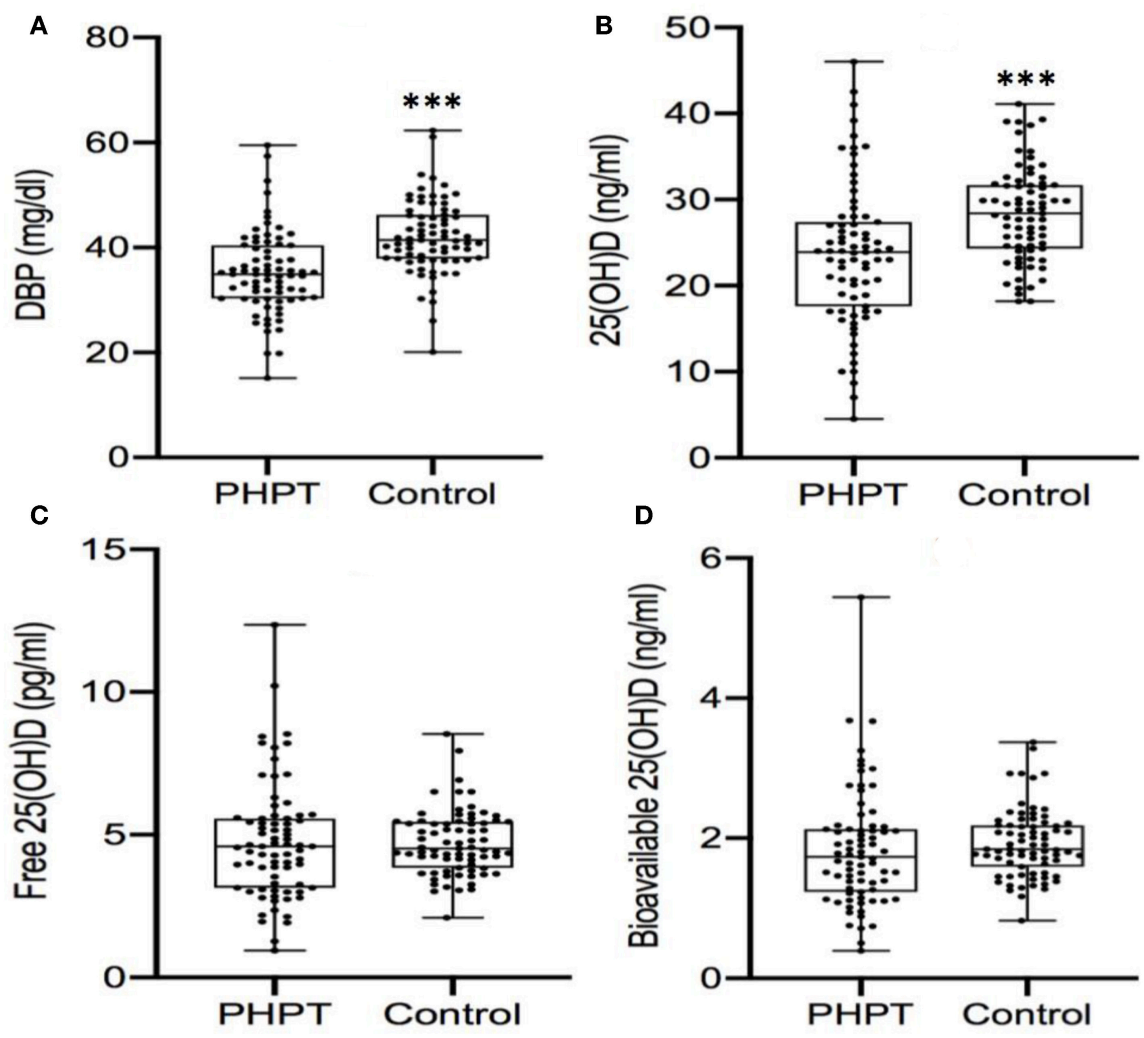
**(A-D)** Comparison of serum levels of DBP and 25 (OH) D between PHPT patients and control subjects. DBP, vitamin D binding protein; 25OHD, 25-hydroxyvitamin D. ^***^P < 0.001.

Seventy-five PHPT patient (23 men, 11 premenopausal women, and 41 postmenopausal women) and 75 control subjects (19 men, 11 premenopausal women, and 45 postmenopausal women) were included in this study. The mean concentrations of calcium, albumin, 25(OH)D, iPTH, and DBP determined in the serum samples from control subjects and PHPT patients are shown in (Table 1). Both total 25(OH)D and DBP levels were about 17% lower in PHPT patients compared to control subjects (*P* < 0.001). There was no significant difference in albumin-bound 25(OH)D, but DBP-bound 25(OH)D was also 17% lower in PTHP patients compared to control subjects (*P* < 0.001). In addition, albumin levels were significantly lower in PHPT patients than in control subjects (*p* < 0.001). There were no significant differences between bioavailable and free 25(OH)D between healthy controls and PHPT patients (Table 1). Comparison of individual 25(OH), DBP, free 25(OH)D, and bioavailable 25(OH) D between healthy controls and PHPT patients were showed in Figure 1.

**Table 1:** Subject characteristics and serum concentrations.

**Variable**	**Control**	**PHPT**	***P*-value**
	***n* = 75**	***n* = 75**	
Age (years)	58.0 ± 8.1	59.3 ± 12.3	0.314
BMI (kg/m^2^)	29.9 ± 2.1	30.6 ± 4.8	0.373
Calcium (mg/dL)	9.4 ± 0.5	11.1 ± 0.6	<0.001
iPTH (pg/mL)	37.9 ± 17.3	140.4 ± 70.5	<0.001
25(OH)D (ng/mL)	28.3 ± 5.4	23.6 ± 8.3	<0.001
DBP (mg/dL)	42.1 ± 7.0	35.2 ± 7.9	<0.001
Albumin (g/dL)	4.5 ± 0.2	4.3 ± 0.3	<0.001
DBP-bound 25(OH)D (ng/mL)	26.4 ± 5.1	21.8 ± 7.7	<0.001
Albumin-bound 25(OH)D (ng/mL)	1.9 ± 0.5	1.8 ± 0.8	0.082
Bioavailable 25(OH)D (ng/mL)	1.9 ± 0.5	1.8 ± 0.8	0.083
Free 25(OH)D (pg/mL)	4.7 ± 1.1	4.7 ± 2.0	0.573

*BMI, body mass index; iPTH, intact parathyroid hormone; 25(OH)D, 25-hydroxyvitamin D; DBP, vitamin D binding protein. Data shows as mean ± standard deviation*.

Levels of DPB (*n* = 150) were positively correlated with total 25(OH)D (*r* = 0.253, *P* < 0.01) but inversely correlated with iPTH (*r* = −0.406, *P* < 0.001) and calcium (*r* = −0.423, *P* < 0.001; Table 2). Levels of iPTH inversely correlated with total 25(OH)D (*r* = −0.418, *P* < 0.001) and bioavailable 25(OH)D (*r* = −0.233, *P* < 0.01; Table 2).

**Table 2:** Spearman correlation coefficients between DBP and other variables (*n* = 150).

	**Age**	**BMI**	**Calcium**	**iPTH**	**25(OH)D**	**Albumin**	**Free 25(OH)D**	**Bioavailable 25(OH)D**
DBP	**−0.210**^**a**^	**−0.192**^**a**^	**−0.423**^**c**^	**−0.406**^**c**^	**0.253**^**b**^	**0.139**^**a**^	**−0.344**^**c**^	**−0.295**^**c**^
Age		0.113	0.076	0.055	0.132	−0.074	**0.301**^**c**^	**0.278**^**c**^
BMI			0.038	0.074	**−0.272**^**c**^	−0.141	−0.121	−0.145
Calcium				**0.751**^**c**^	**−0.292**^**c**^	**−0.218**^**b**^	−0.057	−0.114
iPTH					**−0.418**^**c**^	**−0.312**^**c**^	−0.153	**−0.233**^**b**^
25(OH)D						**0.225**^**b**^	**0.767**^**c**^	**0.786**^**c**^
Albumin							0.092	**0.284**^**c**^
Free 25(OH)D								**0.972**^**c**^

*BMI, body mass index; iPTH, intact parathyroid hormone; 25(OH)D, 25-hydroxyvitamin D; DBP, vitamin D binding protein. Bold means significan*.

a P < 0.05;

b P < 0.01;

c *P < 0.001*.

**Figure 2:**
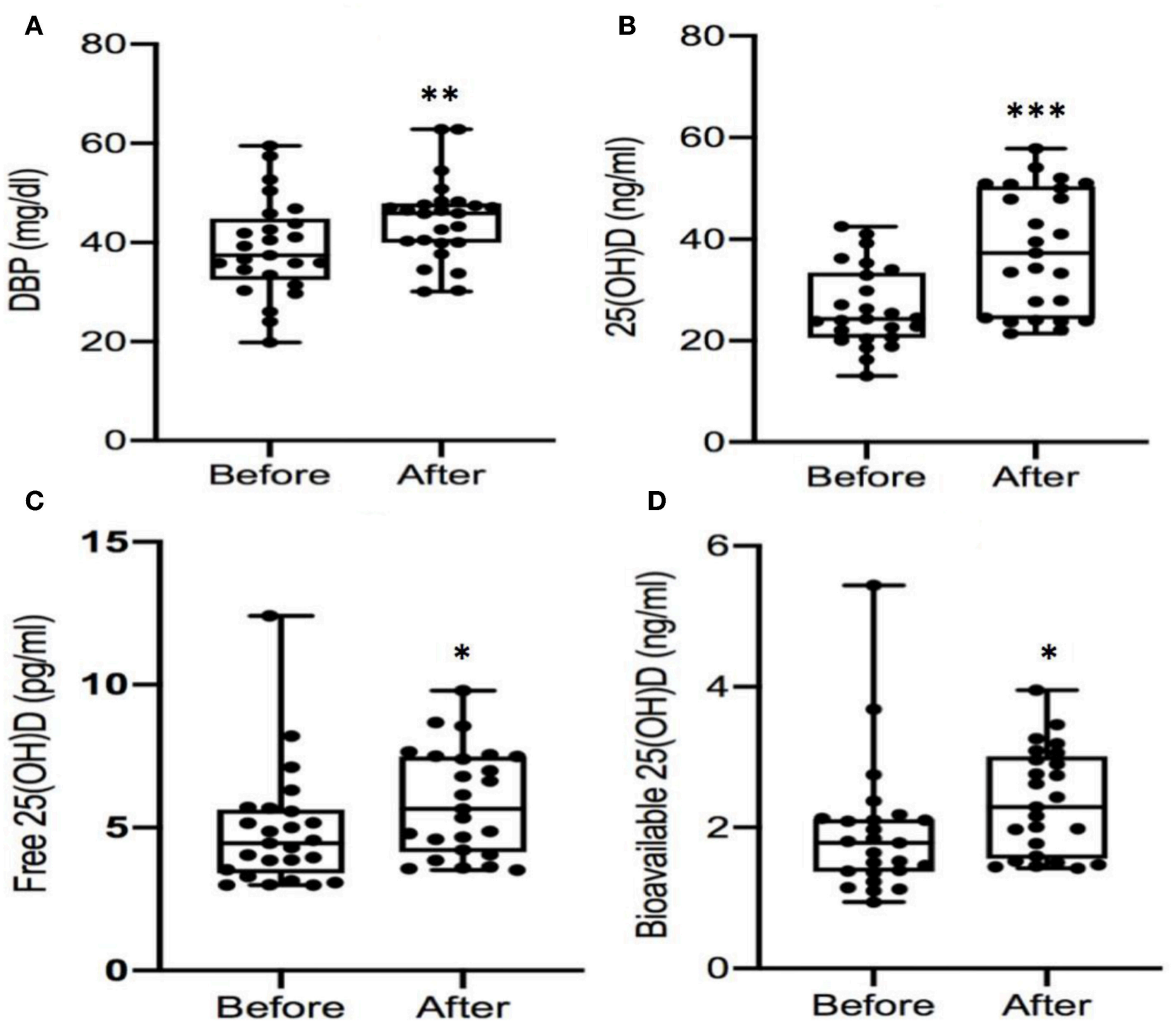
**(A-D)** Comparison of serum levels of DBP and 25(OH)D before and after parathyroidectomy. DBP, vitamin D binding protein; 25OHD, 25-hydroxyvitamin D. ^*^P < 0.05; ^**^P < 0.01; ^***^P < 0.001.

In PHPT patients who underwent parathyroidectomy, serum iPTH, and calcium decreased to normal but DBP levels increased by 15% (*P* < 0.01, Table 3). Serum total 25(OH) D were increased by 43 % (*p* < 0.001) but DBP-bound 25(OH)D also increased by 43% (*P* = 0.001). As a result, there was an attenuated rise in bioavailable (23%, *P* = 0.024) and free 25(OH)D (21%, *P* = 0.021, Table 3). Comparison of individual 25(OH), DBP, free 25(OH)D, and bioavailable 25(OH) D before and after PTX was showed in Figure 2. Multiple regression showed that none of the variables (Ca, PTH or 25(OH)D) predicted the change in DBP after parathyroidectomy (not shown). In addition, there were no predictors for the rise in 25(OH)D due to surgery. Multiple Regression indicated that only the change in albumin predicted change in bioavailable 25(OH)D (*p* = 0.027), but not free 25(OH)D (*p* = 0.122) after parathyroidectomy.

**Table 3:** PHPT profile changes before and after parathyroidectomy.

	**Before**	**After**	***P*-value**
	***n* = 25**	***n*= 25**	
BMI (kg/ m^2^)	31.0 ± 5.6	29.0 ± 4.3	0.127
Calcium (mg/ dl)	11.0 ± 0.6	9.7 ± 0.4	<0.001
iPTH (pg/ ml)	121.5 ± 40.5	44.7 ± 8.2	<0.001
25(OH)D (ng/ ml)	26.4 ± 7.8	37.7 ± 12.1	<0.001
DBP (mg/ dl)	38.9 ± 9.8	44.7 ± 8.2	0.001
Albumin (g/ dl)	4.3 ± 0.9	4.5 ± 0.2	0.046
DBP-bound 25(OH)D (ng/ mL)	24.6 ± 7.3	35.4 ± 11.1	<0.001
Albumin-bound 25(OH)D (ng/ mL)	2.0 ± 0.5	2.36 ± 0.75	0.024
Bioavailable 25(OH)D (ng/mL)	1.94 ± 0.94	2.36 ± 0.74	0.024
Free25(OH)D (pg/mL)	4.89 ± 2.05	5.90 ± 1.9	0.021

*BMI, body mass index; DBP, vitamin D binding protein; iPTH, intact parathyroid hormone; 25(OH)D, 25-hydroxyvitamin D. Data are means ± standard deviation (Paired t-test)*.

## Discussion

The results of our current study demonstrate that PHPT patients have lower serum levels of DBP and total serum 25(OH)D consistent with our previous study (8) and study by Battista et al. (7). In addition, our data confirmed our previous studies that the calculated free or bioavailable 25(OH)D remained unchanged compared with normal control subjects (8). We also showed that PTX increases DBP and DBP-bound 25(OH)D levels. Thus, based on our findings and because supplemental vitamin D raises 25(OH)D, but not DBP (13–15), we suggest that the increased DBP level after PTX might be due to the decreased iPTH or lowered calcium levels after surgery. This supports the hypothesis that DBP is not simply a biomarker of 25(OH)D. Our current results also support the concept that the lower DBP levels in PHPT compared to healthy matched controls may be one of the factors contributing to the low total 25(OH)D levels in PHPT patients. Another possible factor leading to the low total 25(OH)D levels in PHPT patients include the conversion to 1,25 or 24,25 (OH)2D due to elevated iPTH or FGF-23 (6, 16).

Aloia et al. found that black Americans have lower levels of total 25(OH)D but the free 25(OH)D remains relative unchanged by direct measurement of free 25(OH)D (17). Pre-menopausal women have higher serum DBP, estradiol, and 25(OH)D levels than postmenopausal women (18). The calculated free 25OHD was also lower in postmenopausal women than that of control subjects, but to a much lesser degree than total 25OHD (18). In a recent study, Pilz et al. found that women taking estrogen containing contraceptive measures have higher total 25(OH)D but unaltered free 25(OH)D levels by direct measurement (19). The results suggest that total 25(OH)D levels might not be an accurate marker of bioactive vitamin D status in at least a few situations, including black Americans or women taking hormonal contraceptive pill or other clinical situations (5, 20) Bioavailable 25(OH)D may be a better measure of vitamin D status with respect to bone mineral density (BMD) and mineral metabolism, as has been shown in nephrotic syndrome patients (21). Lai et al. found that cirrhosis patients with low albumin had lower DBP, total 25(OH)D, and free 25(OH)D levels and suggest that total 25(OH)D is not accurate marker for vitamin D status in these patients (22). Yu et al. reported that it is bioavailable and free 25(OH)D levels, not total 25(OH), associated with the risk of mortality in Chinese patients with coronary artery disease (23). Our results suggest that the total 25(OH)D levels in PHPT patients may not be a good indicator of vitamin D status before or after surgery since there is a much lower rise in both bioavailable and free 25(OH)D concentrations.

The appropriate management of asymptomatic PHPT still require more evidence from clinical studies (1, 24) despite the guidelines for PHPT have been revised recently (25). There are controversies about vitamin D supplementation in the PTHP patient with low 25(OH) levels. Marcocci et al. reviewed three studies; two demonstrated that vitamin D supplementation had no significant influence on serum and urinary calcium levels, and one study showed no clinical benefit, while in the third study of 27 PHPT patients, 12 patients developed either increased serum calcium levels or increased urine calcium excretion (24). Our data show here that there are no significant differences in free and bioavailable 25(OH)D levels between PHPT patients and control subjects. Given the pre-existing high serum Ca in PHPT patients, we suggest that clinicians should be aware of this treating PHPT patients with vitamin D, especially when using a loading dose of vitamin D supplementation (26).

The DBP concentration is relatively stable throughout life but is altered by gender, menopausal status (10), and genetic backgrounds (27–29). In the current study, we matched PHPT patients and control subjects for these factors. The underlying mechanism for lower DBP concentration in PHPT patients, at least in part, may be explained by the higher iPTH levels inhibition of liver-derived DBP in PHPT patient, a finding that has been reported previously (8) and PTH/PTH-related protein receptor is highly expressed in the liver (30). Conditions such as malnutrition and liver failure might affect DBP, albumin, and other liver-specific protein status (22). Serum DBP concentrations were inversely correlated with iPTH and calcium levels and DBP increased after decreasing iPTH and calcium by parathyroid surgery. It is also possible that a reduced DPB is a compensatory mechanism in PHPT to ensure that under conditions of low total 25(OH)D, there is adequate circulating free or bioavailable 25(OH)D. Also, the mechanism regulating the rise in DBP after parathyroidectomy remains unclear, but it is suggested that studying this population may help to better understand the binding protein and its regulation of normal vitamin D metabolism.

The limitations of the study are the relatively small sample size, including only 25 PHPT patients had 3 months' post-surgery data. Total 25OHD levels were measured by RIA, and not by mass spectrometry which is considered more accurate, but we used internal and external controls to increase accuracy. Another limitation is that this study does not include serum phosphate or FGF-23 levels and the study design cannot confirm a mechanism of low total 25(OH)D or DBP in PHPT. All patients took calcitriol for 1–2 weeks after surgery and advised to take a vitamin D supplement, as standard post-operative clinical care (11) to prevent risk of low serum calcium levels. As a result, this may be another reason for the increase in serum total 25(OH)D at 3 months after parathyroidectomy. Moreover, calculated free 25(OH)D utilize equations that use average binding coefficients for DBP and albumin may not be as accurate as direct measurements (27).

In conclusion, total 25(OH)D and DBP levels are lower in PHPT patients but calculated free 25(OH) remained relatively unchanged. Parathyroidectomy increased DBP and DBP-bound 25(OH)D levels. Further research is required to investigate whether free 25OHD is the better marker of vitamin D status in the PHPT patient.

## Author Contributions

ZS, LM, and CS contributed to recruiting patients, data collection and analysis, and manuscript preparation. ST contributed to Parathyroidectomy and manuscript preparation. SS contributed to experimental design, recruiting control subjects, data analysis and manuscript preparation. XW contributed to experimental design, data analysis and manuscript preparation.

### Conflict of Interest Statement

The authors declare that the research was conducted in the absence of any commercial or financial relationships that could be construed as a potential conflict of interest.
